# Molecular basis of binding between the global post-transcriptional regulator CsrA and the T3SS chaperone CesT

**DOI:** 10.1038/s41467-018-03625-x

**Published:** 2018-03-22

**Authors:** Fei Ye, Fanli Yang, Ruijie Yu, Xi Lin, Jianxun Qi, Zhujun Chen, Yu Cao, Yuquan Wei, George F. Gao, Guangwen Lu

**Affiliations:** 1 West China Hospital Emergency Department (WCHED), State Key Laboratory of Biotherapy and Cancer Center, West China Hospital, Sichuan University, and Collaborative Innovation Center of Biotherapy, Chengdu, Sichuan 610041 China; 2 CAS Key Laboratory of Pathogenic Microbiology and Immunology, Institute of Microbiology, Chinese Academy of Sciences, 100101 Beijing, China; 3 Disaster Medicine Center, Sichuan University, Chengdu, 610041 Sichuan China; 4 Savaid Medical School, University of Chinese Academy of Sciences, 100049 Beijing, China; 5 National Institute for Viral Disease Control and Prevention, Chinese Center for Disease Control and Prevention (China CDC), 102206 Beijing, China; 6 Research Network of Immunity and Health (RNIH), Beijing Institutes of Life Science, Chinese Academy of Sciences, 100101 Beijing, China

## Abstract

The T3SS chaperone CesT is recently shown to interact with the post-transcriptional regulator CsrA to modulate post-attachment signaling in enteropathogenic and enterohemorrhagic *Escherichia coli*. The molecular basis of the CesT/CsrA binding, however, remains elusive. Here, we show that CesT and CsrA both created two ligand binding sites in their homodimers, forming irregular multimeric complexes in solution. Through construction of a recombinant CsrA-dimer (Re-CsrA) that contains a single CesT binding site, the atomic binding features between CesT and CsrA are delineated via the structure of the CesT/Re-CsrA complex. In contrast to a previously reported N-terminally swapped dimer-form, CesT adopts a dimeric architecture with a swapped C-terminal helix for CsrA engagement. In CsrA, CesT binds to a surface patch that extensively overlaps with its mRNA binding site. The binding mode therefore justifies a mechanism of CsrA-modulation by CesT via competitive inhibition of the CsrA/mRNA interactions.

## Abstract (web summary)

CesT is a type III secretion system chaperone that interacts with the post-transcriptional regulator CsrA, which is important for the modulation of post-attachment signaling in enteropathogenic and enterohemorrhagic *Escherichia coli*. Here the authors present the structure of the CsrA/CesT complex and propose a mechanism for CsrA-modulation by CesT.

## Introduction

The pathogenic *Escherichia coli* (*E. coli*) strains of enteropathogenic and enterohemorrhagic *E. coli* (EPEC and EHEC) can cause severe diseases in humans, resulting in the characteristic attaching and effacing (A/E) lesions^1,2^. These pathogens has evolved to acquire a wide variety of virulence genes, among which those encode for a type III secretion system (T3SS) and the related proteins are shown to play central roles in the colonization and A/E pathogenesis of the bacteria^3^. T3SS is widely distributed among Gram-negative pathogens and functions to translocate diverse bacterial effector proteins into host cells^4^. In EPEC and EHEC, a battery of up to 40 effector proteins are delivered via T3SS, leading to not only enterocyte damages but also subversions of multiple cellular signaling pathways^3^. It was recently shown that T3SS also participates in host cell sensing and post-attachment remodeling of gene expressions. The process was demonstrated to be mediated by the T3SS-specific chaperone CesT antagonizing the post-transcriptional regulator CsrA^5^.

CesT was initially identified as the chaperone for E. *coli* secreted protein Tir (translocated intimin receptor)^6^. It was later shown that CesT also interacts with a variety of bacterial effectors (e.g., Map, NleA, NleG, NleH, NleH2, EspF, EspG, EspH, and EspZ) and is therefore better characterized as a multicargo T3SS chaperone^7–9^. In addition to effector binding, CesT is also shown to play a role in effector secretion, mediating hierarchical protein translocations^10^. It is notable that Infection studies in both cell line and animal models have demonstrated that CesT is essential for efficient host colonization^6,7^. The chaperone protein is, therefore, a key T3SS player involved in the pathogenesis of EPEC and EHEC.

As with other T3SS chaperones, CesT exists as stable homodimers in solution^11,12^. A previous study has reported the atomic structure of CesT, which shows a dimeric architecture with a swapped N-terminal domain^11^. Nevertheless, the biological relevance of this dimer form remains to be investigated, since a majority of the homologous T3SS chaperones utilize an unswapped dimer for effector binding^13–18^. It is notable that it has been proposed that CesT, in the absence of a domain-swap event, might also form an unswapped dimer similar to other T3SS chaperones^11^. A later NMR study also indicated the presence of such a dimer form in solution^12^.

The Csr/Rsm system (for carbon storage regulator or repressor of secondary metabolism) is a widespread regulatory system that is present in abundant bacteria species^19–21^. Functionally, the system can mediate post-transcriptional regulation of gene expression and modulate multiple physiological processes, including central carbon metabolism, secondary metabolite metabolism, motility, biofilm formation, virulence factor production, and etc.^19–21^. A central player in this system is the CsrA/RsmA protein which is able to bind the ribosome-binding site (RBS) of target messenger RNAs (mRNAs) and thereby repress translation initiation^22,23^. The activity of CsrA is further modulated by small non-coding RNAs (sRNAs, e.g., CsrB/C and RsmX/Y/Z^24,25^), which can competitively antagonize the binding of CsrA to mRNA and relive the translation repression^26,27^.

CsrA and its homologous proteins form highly conserved domain-swapped homodimers^28–30^. The mechanism of CsrA-mediated translational modulation has been illustrated with the *Pseudomonas fluorescens* (*P. fluorescens*) CsrA-homolog of the RsmE protein. The complex structure of RsmE bound with an RBS RNA-fragment derived from the *hcnA* gene revealed two RNA binding sites in the protein dimer, making optimal contacts with an ^A^/_U_CANGGANG^U^/_A_ sequence motif^23^. Later studies showed that sRNAs can engage the same RNA binding site in RsmE, thereby competing against the RsmE/mRNA interactions^26,27^.

In addition to the sRNA-dependent regulation, the CsrA activity can also be modulated by proteinaceous ligands. A representative example of such case is the FliW protein, which has been shown to interact with CsrA to modulate the flagellin homeostasis in bacteria such as *Bacillus subtilis*^31,32^. The CesT protein is a newly identified CsrA ligand in *E. coli*. Noted the central roles of CesT in T3SS and of CsrA in the global regulation (over 15% of all mRNAs in *E. coli*^33^) of gene expression, illustration of the CesT/CsrA recognition basis will be a key issue in understanding the pathogenesis of EPEC and EHEC.

In this study, we delineate the molecular basis of the binding between CesT and CsrA via comprehensive biochemical and structural studies. We show that the intimate interactions between CesT and CsrA require both proteins to first assemble into a dimer. CesT adopts a dimeric architecture with a swapped C-terminal helix, which has not been observed before, for CsrA engagement. With an extensively overlapped site involved in binding to both CesT and RNA, we further show that CesT modulates the CsrA activity via competitively antagonizing its binding to mRNAs.

## Results

### A recombinant CsrA dimer with a single CesT binding site

**Fig. 1:**
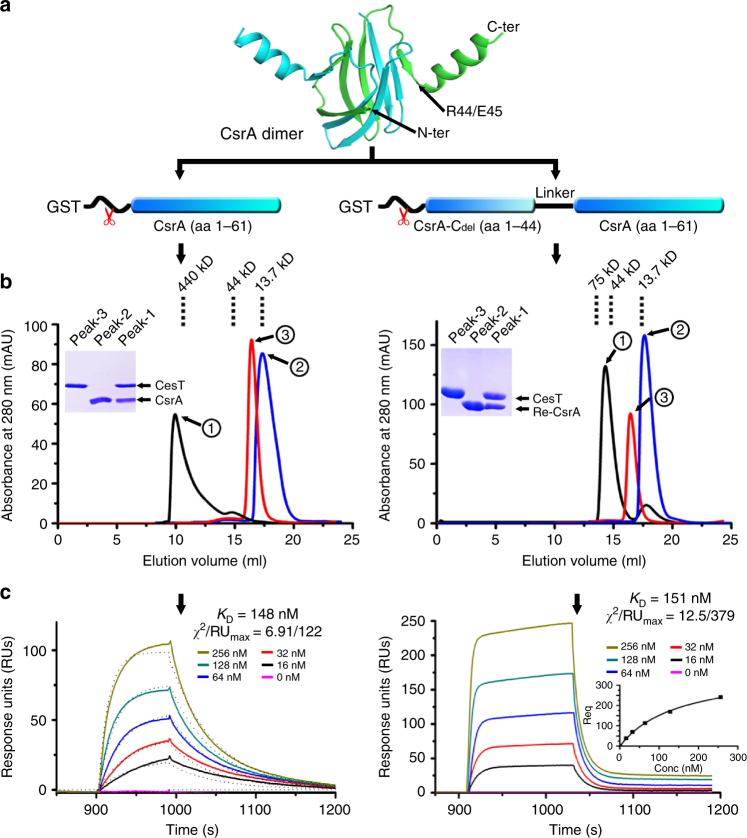
Biochemical characterization of the bindings for the CesT/CsrA and CesT/Re-CsrA pairs. **a** A schematic figure showing the construction and expression strategies for the native CrsA and Re-CsrA proteins. The native domain-swapped CsrA-dimer structure (PDB code: 2BTI) is shown in cartoon representation to highlight the β-barrel core and the terminal α-helix which are bordered at R44/E45. Re-CsrA is a recombinant CsrA dimer, composed of a C-helix-deleted CsrA (CsrA-C_del_) connecting via a linker to a full length CsrA. Both CsrA and Re-CsrA are initially expressed as GST-fusion proteins, which are then enzymatically digested to remove the GST-tags. **b** Analyses of the CesT/CsrA and CesT/Re-CsrA complexes by analytical gel filtration. The 280-nm absorbance curves and the SDS-PAGE migration profiles of the indicated proteins are shown. Dashed lines indicate the standard molecular weight markers. **c** An SPR assay characterizing the real-time binding kinetics of CsrA and Re-CsrA to immobilized CesT. The recorded profiles are shown. The affinity is calculated using the Bivalent-Analyte model for the native CsrA and the steady-state affinity model for Re-CsrA, respectively. The calculated-fit curve is shown as dashed lines for CsrA and as a built-in panel for Re-CsrA. The goodness of fit between the experimental data and the model algorithm is also highlighted by the low *χ*^2^/RU_max_ values (<10%)

According to previous studies^11,12,28^, both CesT and CsrA exist as stable homodimers in solution. These results remind us of the possibility that two identical ligand binding sites could be present in both CesT and CsrA dimers. We therefore first investigate the binding features between the two proteins via analytical gel filtration chromatography. CsrA was initially expressed as a GST-fusion protein and then enzymatically digested to remove the fusion-tags (Fig. 1a); while CesT was expressed as a 6xHis-tagged protein. Both proteins were individually prepared to homogeneity and then mixed for in vitro binding analyses. As expected, stable CesT/CsrA complex forms in solution. The resultant complex species is mainly eluted in the void peak with an estimated molecular weight of more than 440 kDa and exhibits high heterogeneity (Fig. 1b). Noted that CesT and CsrA dimers are of ~35.4 kDa and ~13.7 kDa respectively, the result is therefore consistent with our expectations that both CesT and CsrA dimers contain two ligand binding sites, leading to irregular protein aggregations upon complex formation. We further determined the real time binding kinetics between CesT and CsrA via surface plasmon resonance (SPR). The observed profile revealed typical slow-on/slow-off binding characteristics, and fits well with the Bivalent-Analyte model which describes the binding of a bivalent protein (in the current case the CsrA dimer) to an immobilized ligand (in the current case CesT) (Fig. 1c). The affinity (*K*_D_) of the first ligand binding site in CsrA to CesT was calculated to be ~148 nM.

To obtain homogeneous CesT/CsrA protein complexes that are suitable for structural study, we set out to disrupt the complex aggregations arising from binding of the two bivalent proteins. It is notable that CsrA and its homologous proteins in other bacteria contain an extended C-terminal α-helix which has been shown in previous reports to play pivotal roles in ligand binding^23,26^. We therefore hypothesized that this terminal helical component, which extends from E45 to Y61 (Fig. 1a), is also involved in engaging CesT. To verify this hypothesis, we conducted a GST-pulldown experiment with either the wild-type (WT) CsrA or a CsrA-variant (CsrA-C_del_, residues M1-R44) which lacks the C-terminal helix. While the WT protein efficiently pulldowns CesT, CsrA-C_del_ completely lost its CesT binding avidity (Supplementary Fig. 1). The result enabled us to make a new CsrA construct that reserves only a single CesT binding site by linking CsrA-C_del_ with full-length CsrA via a ten-residue linker (GGGGSGGGGS) (Fig. 1a). As expected, the resultant recombinant protein (denoted Re-CsrA hereafter) remains competent in CesT engagement and forms stable complex with CesT in solution as with WT CsrA (Fig. 1b). In contrast to the WT protein-complex, however, the CesT/Re-CsrA complex behaves as homogeneous protein species with an estimated molecular weight of ~60 kDa, coinciding well with two Re-CsrA (~12.5 kDa) molecules binding to one CesT dimer. The interactions between Re-CsrA and CesT were further analyzed via SPR. In contrast to WT CsrA, the recombinant protein binds CesT with a fast-on/fast-off mode (Fig. 1c). Despite of the altered binding kinetics (which is commonly observed in the ligand/receptor interactions when the interface residues are mutated^34–36^), the affinity between Re-CsrA and CesT, which is calculated to be ~151 nM, is in good accordance with that observed for the WT proteins. The recombinant protein therefore represents a good candidate for structural investigations on the molecular basis of CesT/CsrA recognition.

### The complex structure between CesT and Re-CsrA

**Fig. 2:**
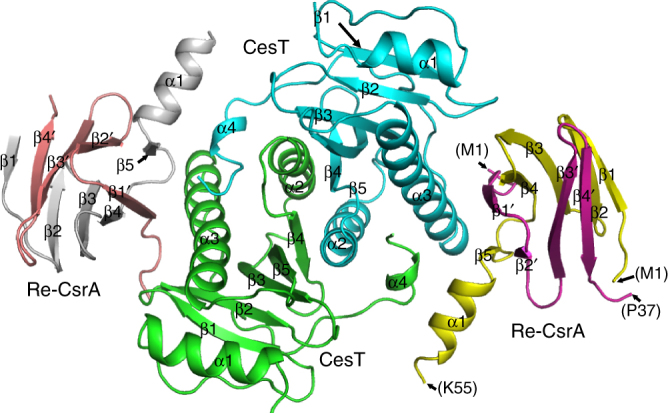
The overall structure of the CesT/Re-CsrA complex. Two Re-CsrA molecules symmetrically bind to the CesT dimer. The secondary structure elements are labeled. In Re-CsrA, the prime symbols indicate the elements of the CsrA-C_del_ moiety. The terminal residues of the CsrA-C_del_ and the full-length CsrA moieties are marked with letters in parentheses

Inspired by the high homogeneity of the CesT/Re-CsrA complex, we used this protein species for crystal screening and managed to collect a dataset of 2.3-Å resolution. The structure was solved by molecular replacement and refined to *R*_work_ = 0.233 and *R*_free_ = 0.251, respectively (Table 1). The final model contains, in the crystallographic asymmetric unit, two CesT/Re-CsrA complexes which are essentially of the same structure. Superimposition of the two complexes reveals a root mean square deviation (r.m.s.d.) of 0.713 Å for all the equivalent Cα atoms. As expected, each complex is composed of two Re-CsrA molecules and one CesT dimer (Fig. 2). For Re-CsrA, clear electron densities could be traced for residues M1-P37 of the CsrA-C_del_ moiety and residues M1-K55 of the following full-length CsrA entity. These amino acids assemble into a compact β-barrel core with one terminal helix extending out into the bulk solvent. Overall, the Re-CsrA structure is quite similar to that of the swapped dimer of native CsrA. Superimposition of the two structures revealed both well-aligned core-barrel strands and the terminal helix. The secondary structure components of Re-CsrA (strands β1′–β4′ in CsrA-C_del_, β1–β5 and α1 in full-length CsrA) were therefore labeled such that CsrA-C_del_ was viewed as an equivalent to the second protomer of the native dimer, highlighted with the prime symbols. Without helix α1′, Re-CsrA also lacks traceable electron densities for the β5′ strand due to flexibility, which could be otherwise clearly observed in the native CsrA dimer structure (Supplementary Fig. 2). It is also noteworthy that Re-CsrA uses its peripheral side-patch, created by strands β1′, β4, β5, and helix α1, for CesT binding (Fig. 2). The structure therefore in turn verifies our analytical gel filtration results, demonstrating that the recombinant CsrA protein indeed retains a single but integral CesT binding site.

**Table 1:** Data collection and structure refinement statistics

CesT/Re-CsrA	
*Data collection* ^a^	
Space group	C2
Cell dimensions	
* a*, *b*, *c* (Å)	252.8, 52.6, 93.4
* α*, *β*, *γ* (°)	90.0, 96.7, 90.0
Wavelength (Å)	0.97775
Resolution (Å)	50–2.3 (2.38–2.30)
*R*_merge_	0.072 (1.308)
*R*_pim_	0.030 (0.557)
CC_1/2_	0.997 (0.877)
*I*/sig*I*	23.0 (1.25)
Completeness (%)	99.8 (99.8)
Redundancy	6.7 (6.5)
*Refinement*	
Resolution (Å)	49.35–2.30
No. reflections	54651
Completeness (%)	98.8
*R*_work_/*R*_free_	0.233/0.251
No. atoms	
Protein	7830
Ligand/ion	0
Water	138
*B*-factors	
Protein	87.03
Ligand/ion	
Water	67.49
R.m.s. deviations	
Bond lengths (Å)	0.003
Bond angles (°)	0.579
Ramachandran plot (%)	
Favored region	96.40
Allowed region	3.60
Outlier region	0

Values in parentheses are for the highest-resolution shell

^a^ A single crystal was used to collect the data

For CesT, two protomer molecules of the same fold assemble into a tight dimer. Each protomer is topologically arranged in an αβββαββαα manner, structurally folding into a five-stranded antiparallel β-sheet (β1–β5) in the center which is further wrapped by three α-helices (α1–α3) at peripheral. The fourth helix (α4), however, is linked to α3 via a long inter-loop and extends to the vicinity of the other CesT molecule in the dimer (Fig. 2). On the whole, the two CesT protomers symmetrically pack against each other around the α2–β4 helix-strand motif, leading to a dimer with typical two-fold non-crystallographic rotation symmetry. Accordingly, the bound Re-CsrA molecules are related by the same two-fold axis, recognizing the same surface components in CesT (Fig. 2).

### A CesT dimer with a swapped C-terminal helix

**Fig. 3:**
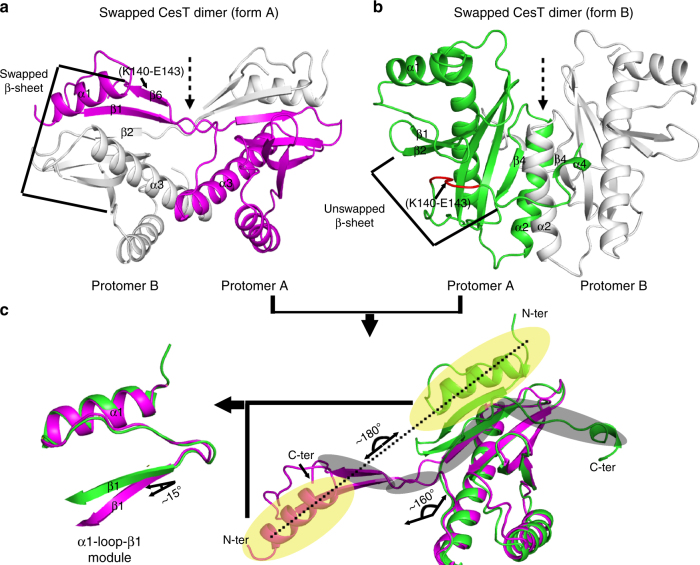
Two distinct domain-swapped dimeric architectures of CesT. **a** The form A dimeric architecture (based on PDB code: 1K3E). **b** The form B dimeric architecture (based on the complex structure solved in the current study). Those secondary structure elements referred to in the text are labeled. The swapped β-sheet in the dimer form A and the unswapped β-sheet in the dimer form B are highlighted. Residues K140-E143, which adopt a rigid strand conformation in dimer form A but exhibit a loop structure in dimer form B, were marked and colored red in **b** for clarity. The two-fold rotation axes are shown as dashed arrows. **c** Comparison of the single protomer structures derived from the two forms of CesT dimers. The N-terminal α1-loop–β1 module and the extended C-terminal motif are shaded in yellow and in gray, respectively. The profound orientation-divergences are marked. The N-module is further superimposed to highlight their overall similar folds but a slight orientation-difference in strand β1

A previous study on CesT reveals that the molecule, unlike the other bacterial T3SS chaperones as exemplified by the SigE molecule of *Salmonella enterica*, exhibited a unique domain-swapped dimeric architecture^11^. In this dimer structure (denoted dimer form A hereafter), the N-terminal α1 helix, β1 strand, and their intervening loop form a discrete module, intertwining with the other protomer such that its β1 strand pairs with strand β2 from the other CesT molecule. In addition, the C-terminal residues K140-E143 fold into an extra strand (β6), aligning in an antiparallel manner with β1 on the other side (Fig. 3a). In such a dimer, the long α3 helices from the two protomers further symmetrically pack against each other. This domain-arrangement mode therefore created a CesT dimer with a two-fold rotation symmetry around an axis that locates in between the two swapped β-sheets and vertically passes through the packed α3 helices (Fig. 3a). It is also notable that due to strand-swapping, the dimer is predominantly stabilized by the main-chain hydrogen bond contacts among adjacent β-strands with hydrophobic interactions of less profoundness contributed by the apolar residue side-chains.

In the complex structure of Re-CsrA bound to CesT, however, the chaperone molecule adopted a quite different dimeric fold (denoted dimer form B hereafter). The N-terminal α1-loop–β1 module, instead of being swapped into the other protomer, remains unswapped and pairs with strand β2 of its own molecule. Without the swapped β1 strand, the C-terminal residues K140-E143 form a loop in our structure, which were otherwise of rigid strand conformation (β6) in the form A dimer described above. Overall, the CesT molecules assemble into a dimer around the α2–β4 motif such that the α2 helices in the two protomers are aligned with each other in an almost parallel manner. The dimer structure is therefore symmetrically related with a two-fold rotation axis which vertically passes through the packed α2–β4 components (Fig. 3b).

We further compared the single protomer structures derived from the two forms of CesT dimers. As expected, the most profound structural variations were observed in their terminal components: the N-terminal α1-loop–β1 module and the extended C-terminal motif. The former flipped ~180° from swapping out for inter-molecule β-paring in dimer form A to folding back for intra-molecule β-pair interactions in dimer form B (Fig. 3c); while the latter similarly experienced a significant conformational change, reorienting for ~160° from interacting as a β6 strand (in dimer form A) with β1 to spreading as an extended loop and an α4 helix (in dimer form B) over the molecule surface (Fig. 3c). It is also notable that with profound divergence in its orientation, the α1-loop–β1 module retains essentially the same structure except that its β1 strand exhibited a slight (~15°) orientation-difference relative to helix α1 between the two dimer forms (Fig. 3c).

On the whole, the architecture of the form B CesT dimer is quite similar to that observed in the unswapped salmonella SigE molecule (Supplementary Fig. 3a, 3b). In both dimers, the two protomers assemble around the α2 helix and β4 strand, forming a core dimeric interface in which multiple apolar and aromatic residues created a strong hydrophobic center. The CesT dimer, however, was further clenched at peripheral by the extended C-terminal motif. A most important stabilizing contact was contributed by the α4 helix, which protrudes into the otherwise exposed groove formed by helices α2 and α3 in the other protomer and presents several residues (especially Y153) for mainly apolar inter-molecule interactions (Supplementary Fig. 3c). It is unexpected that the C-terminal part of CesT forms a helical structure and extends exclusively into the other protomer of the dimer. It is notable that other T3SS chaperones, such as SycT from *Yersinia enterocolitica*, also contains an extended C-terminal loop which interacts with the β2–β3 inter-loop from the other molecule of the dimer^37,38^. Nevertheless, CesT extends its C-terminal helix into the other molecule to a much greater extent, providing much stronger and more intimate inter-molecule interactions than those observed for SycT (Supplementary Fig. 3d). Such dimeric arrangement with a swapped C-terminal domain seems unique which has not been observed in the other T3SS chaperone structures reported previously^13–18^. It is also noteworthy that the α2–α3 helices of one CesT molecule also in turn stabilized the swapped α4 helix, thereby preparing it for contacts with CsrA (see Results below).

### Structural basis of the binding between CesT and CsrA

**Fig. 4:**
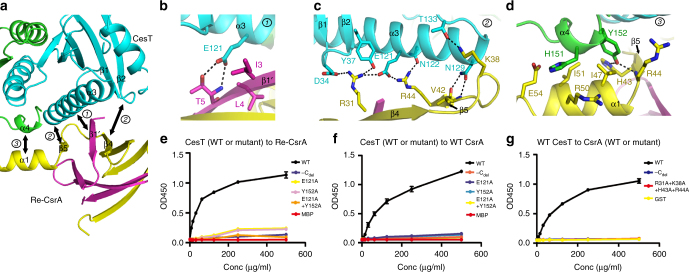
The atomic binding details between CesT and Re-CsrA. **a** An overview of the binding interface. Based on the protomer-affiliations (to either the parental molecule of the dimer or the second protomer) of the engaging components, the inter-molecule CesT/CsrA interactions are allocated to three binding patches which are further illustrated in **b**–**d** for amino acid interaction details. **b** Interactions contributed by the second CsrA protomer contacting the parental CesT molecule. **c** Interactions contributed by both the CsrA and CesT parental molecules. **d** Interactions contributed by the parental CsrA contacting the second CesT protomer. The engaging components and the interface residues are shown and labeled. Dashed lines indicate hydrogen bonds or salt bridges. **e**–**g** An ELISA assay verifying the roles of the identified key interface residues in CesT/CsrA interactions. Serial dilutions of the indicated CesT (WT or mutant) proteins were tested for their binding to immobilized CsrA or Re-CsrA (WT or mutant) proteins. The recorded absorbance at 450 nm is plotted and shown. All the results are expressed as means ± standard deviations (SD) from three independent experiments. **e** Binding of WT or mutant CesT to Re-CsrA. **f** Binding of WT or mutant CesT to native CsrA. **g** Binding of WT CesT to CsrA of WT or mutant forms

The atomic binding details between CesT and CsrA were then systematically investigated based on the complex structure solved in this study. Overall, Re-CsrA utilizes its intact CsrA-dimer part composed of helix α1, strands β1′, β4, β5, and their intervening loops for CesT engagement, contacting mainly the β1–β2 interloop, the long α3 helix, and the swapped terminal helix α4 (from the other protomer) in the chaperone dimer (Fig. 4a). In total, these components buried a surface area of 836.2 Å^2^ in Re-CsrA and 893.9 Å^2^ in the CesT dimer, respectively. Along this extended interface, the inter-molecule amino acid interactions can further be allocated to three binding patches (Patch1, 2, and 3) as to the protomer affiliations (to either the parental molecule of the dimer or the second protomer) of the engaging components. The first patch involves Re-CsrA strand β1′ and CesT helix α3, representing interactions contributed by the second CsrA protomer with the parental CesT molecule (Fig. 4a). The former presents three residues I3, L4, and T5 to pack against CesT E121, providing both hydrogen bond and apolar carbon–carbon interactions (Fig. 4b). In contrast to Patch1, Patch2 is much more extended and consists of multiple components from the CsrA and CesT parental molecules (Fig. 4a). A series of charged and hydrophilic residues were observed to locate in this second patch, forming a strong polar (hydrogen bond and/or salt bridge) contact network. These include the Re-CsrA residues R31 located in strand β4, K38 in the β4–β5 inter-loop, and V42 (with its main-chain groups) and R44 in strand β5 interacting with the CesT amino acids D34 in the β1–β2 intervening loop, Y37 in the β2 strand, and E121, N122, N129, T133 in the α3 helix and its following loop (Fig. 4c). For Patch3, it is mainly the parental CsrA contacting the second CesT protomer, involving strand β5 and helix α1 in Re-CsrA and the swapped α4 helix in CesT (Fig. 4a). The α4-residues, including H151 and Y152, were observed to protrude out for Re-CsrA interactions, stacking over CsrA residues H43, R44, I47, R50, I51, and E54 to provide dozens of hydrophobic contacts via their apolar carbon atoms. In addition, Y152 of CesT also forms two hydrogen bonds with CsrA H43 and R44, respectively (Fig. 4d). The defined amino acid interactions therefore revealed a CesT/CsrA binding mode which is mediated mainly by the polar interactions among hydrophilic interface residues. It is also notable that the full-capacity binding between CesT and CsrA requires both proteins first assembling into a dimer.

To verify the observed binding mode, a series of mutant CesT and CsrA proteins were prepared. Via CD spectroscopy and comparative analytical gel filtration, we first showed that all the mutant proteins are properly folded and remain existing as dimers in solution (Supplementary Fig. 4a, 4b). The proteins were then tested for their ligand binding avidities via ELISA. For CesT, deletion of the C-terminal α4 helix (CesT-C_del_) and individual or simultaneous substitution of the key interface residues E121 and Y152 with alanine all dramatically jeopardized its ability to engage CsrA. While WT CesT potently binds to both native CsrA and Re-CsrA, the CesT mutants only show a very weak binding at the highest concentration tested (500 µg/ml) (Fig. 4e, f). Reciprocally, the four basic interface-residues (R31, K38, H43, and R44) in CsrA were mutated (to alanine) and the resultant mutant protein was tested for its interaction with WT CesT. As expected, this tetra-mutant CsrA protein completely lost its CesT-binding capacity, as with the C-terminal deletion variant CsrA-C_del_ (Fig. 4g). In summarization, these results are in good accordance with the atomic binding details defined in the complex structure, in turn verifying the observed CesT/CsrA interaction mode.

### Mechanism of CsrA modulation by CesT

It is notable that CsrA, as a global post-transcriptional regulator, exerts its regulatory function by binding specifically to the mRNA RBS sequence, thereby leading to translational repression^22^. The atomic binding mode between CsrA and the RBS RNA has been structurally investigated with the pseudomonas CsrA homolog of RsmE^23^. Although the structure of *E. coli* CsrA bound with RNA remains unavailable thus far, the *E. coli* CsrA and *P. fluorescens* RsmE share high sequence identity and conserved structural fold. In addition, previous mutagenesis studies identified two regions in *E. coli* CsrA (residues 2–7 and 40–47) that are important for its RNA binding avidity in vivo^39^. These two regions fall in good accordance with the structural observations in the RsmE/RNA complex structure. We therefore believe *E. coli* CsrA shares a similar RNA binding mode to that of RsmE.

**Fig. 5:**
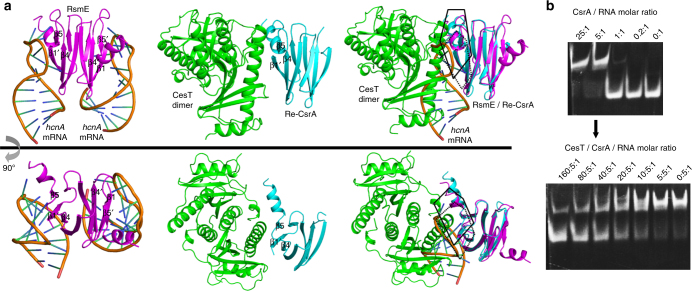
Competition of the CsrA/mRNA binding by CesT. **a** Side-by-side views of the structures of RsmE bound with *hcnA* mRNA (left), Re-CsrA bound with CesT (middle), and the superimposition of the two structures (right). Those shown in the top panel are clockwise rotated for about 90° around a horizontal axis to yield the structures exhibited in the bottom panel. The RsmE/CsrA structural elements involved in binding to both mRNA and CesT are highlighted and labeled. The identified RNA-binding site in RsmE and the CesT-binding site in Re-CsrA are encircled with dashed and solid lines, respectively. Clearly shown is that the RNA binding site in RsmE and the CesT binding site in CsrA extensively overlap with each other. **b** An RMSA experiment characterizing the CsrA repression by CesT. An RNA molecule derived from *nleA* 5′UTR was utilized as the RNA probe for CsrA binding. The resolved native TBE-PAGE gels are shown. Top panel: WT CsrA mixing with RNA at the indicated molar ratios. Bottom panel: pre-incubated CsrA/RNA (at 5:1 molar ratio) mixture with different concentrations of CesT

In such a context, we compared the structure of CesT/Re-CsrA with that of RsmE bound with an 5′-^A^/_U_CANGGANG^U^/_A_-3′ RNA (corresponding to the RBS sequence of the *hcnA* gene, PDB code: 2JPP) to learn the possible CsrA-modulation mechanism by CesT. In the RsmE dimer, two RNA moieties individually bound to the β1′/β4/β5 and its symmetrical β1/β4′/β5′ barrel-patches, defining two identical RNA binding sites (Fig. 5a). The CesT molecule also engages extensively with the β1′, β4, and β5 strands in Re-CsrA (Fig. 5a). Superimposition of the two structures on the Re-CsrA/RsmE proteins revealed an extensively overlapped region between the RNA binding site in RsmE and the CesT binding site in CsrA (Fig. 5a). The binding of CesT to CsrA should therefore competitively inhibits the engagement of CsrA to mRNA, thereby relieving the CsrA-mediated translation repression. To further demonstrate the inhibition of CsrA/mRNA interactions by CesT, we performed an RNA-based motility shift assay (RMSA) using a previously reported RNA molecule derived from the 5′UTR of the *nleA* transcript with the sequence of 5′-CACUAAUAAUAUCAAUGGAUUGAUAUUAUUAAUG-3′^5^. While CsrA is able to effectively bind the RNA molecule, addition of CesT indeed antagonizes the binding of CsrA to RNA (Fig. 5b). Similar results have also been observed in a previous study using the GST fusion protein of CesT^5^.

It is also notable that CesT and CsrA, each of which contains two ligand binding sites in their homodimers, could polymerize in solution (Fig. 1b). In order to investigate, whether polymerization of the CesT/CsrA complex could contribute to CsrA repression by CesT, we performed a comparative RMSA experiment using the CsrA WT + C_del_ mixture (WT CsrA mixing with CsrA-C_del_ in a 1:1 molar ratio) and Re-CsrA, which were first pre-incubated with RNA and then compared for the inhibition efficiencies of the CsrA/RNA interactions by CesT in parallel. In this testing system, the CsrA WT + C_del_ mixture could create equal amounts of the individual intact (containing the C-terminal helix) and the incomplete (lacking the C-terminal helix) ligand-binding sites to the Re-CsrA fusion protein of the same molar quantity. The CesT/CsrA polymerization, however, would only occur in the former case where WT CsrA is present. While the WT + C_del_ mixture is similarly capable of binding to the RNA molecule as Re-CsrA, the inhibition of CsrA/RNA binding by CesT with WT + C_del_ (in which case CesT would polymerize with CsrA) is apparently more effective than that with Re-CsrA (in which case CesT would form uniform complexes with Re-CsrA) (Supplementary Fig. 6). These results therefore revealed an interesting notion that the CesT/CsrA polymerization could benefit the effective repression of CsrA by CesT.

## Discussion

T3SS is an effector-translocation system that plays important roles in the virulence of a wide spectrum of pathogenic gram negative bacteria^4^. In the A/E pathogens, including EPEC, EHEC, and the murine-specific *Citrobacter rodentium* (CR), a recent study showed that the T3SS also functions to trigger post-transcriptional remodeling of gene expression upon cell attachment by modulating the CsrA activity via the T3SS specific chaperone of CesT^5^. Delineation the molecular basis of CesT/CsrA recognition, which links T3SS with the post-attachment signaling in bacteria, is therefore of vital importance in understanding the pathogenesis of these pathogens. In this study, we systematically investigated the CesT/CsrA binding features using the proteins derived from EPEC and have atomically characterized their interface residues involved in the protein engagement. It is notable that these amino acids are absolutely conserved among EPEC, EHEC, and CR (Supplementary Fig. 5a, 5b). These bacteria therefore should share a common CesT/CsrA binding mode as illustrated with the EPEC protein pair.

The canonical function of CesT is to facilitate the translocation of a wide variety of effector proteins from *E. coli* to host cells via T3SS^6–9^. In bacteria, prior to effector-translocation, CesT would form stable complexes with its effectors. It is notable that the CrsA modulation mediated by CesT occurs only after the attachment of *E. coli* to host cells^5^. This phenomenon is highly indicative of a competitive binding between the effector molecules and CsrA to CesT. As a muticargo chaperone, CesT was initially identified as an intrinsic binding partner for the Tir molecule^6^. Previous studies have shown that an amphipathic α-helical region that corresponds to helix α3 in the CesT structure is intimately involved in Tir binding^40^. This α3 helix, according to the complex structure solved in this study, also participates extensively in the interactions with CsrA. The observation seems to justify a competitive relationship between CsrA and Tir.

Although the structures of CesT bound with Tir or with any other effector proteins remain unavailable thus far, the chaperone/effector interactions have been atomically studied in several other gram negative bacteria^13,14^. These structures revealed a common binding mode such that a major binding interface involves an unfolded linear epitope (denoted the β-motif) in the effector forming an intermolecular β-sheet with the chaperone. The chaperone/effector binding, such as in the SipA/InvB complex of *Salmonella*^13^, could be further stabilized by a folded globular effector-domain which interacts with chaperone mainly via hydrophilic interactions. It is notable that CsrA remains in a fully folded state and relies on an extensive polar-contact network for CesT binding. The binding mode parallels the CesT/CsrA interactions with, but only with, the globular protein–protein interactions in the chaperone/effector pairs. This could indicate a priority for the CesT/effector engagement over the interactions between CesT and CsrA. It is interesting that a previous study demonstrated a hierarchical effector-delivery mode in which the expression and secretion of Tir are prior to the efficient secretion of other effectors^10^. We believe the CesT/CsrA engagement should follow the same hierarchical pattern such that delivery of Tir and possibly also other effector proteins into the host cell-cytosol releases CesT for CsrA binding.

Among typical T3SS chaperones, CesT is unique in terms of its N-domain swapped dimer (form A) structure^11^. It should be noted that an alternative dimer form (resembling the dimer form B observed in our structure) can be reconstructed in that N-domain swapped structure, except that the N-terminal α1-loop–β1 module is from a different molecule in the crystal. It is therefore proposed that CesT might also form dimers similar to other unswapped T3SS chaperones in the absence of a domain-swap event^11^. In the current study, we provided solid structural evidence, with the form B dimeric architecture observed in our structure, that such a dimer form indeed exists and is responsible for CsrA engagement. It should be noted that with distinct dimer-assembly patterns, only the dimer form B, but not form A, is able to build integral CsrA binding sites in CesT. In support of this, a previous NMR investigation on CesT also indicates the presence of such a dimer form in solution^12^. In addition to binding to multiple effectors as a universal chaperone, CesT was also shown to play important roles in effector secretion^7,10,41^. It is noteworthy that the ability to facilitate secretion by CesT is allocated to the distant C-terminal region of the protein, which is proposed to exhibit a distinct α-helical structure^41^. Consistent with these previous reports, our structure indeed shows that the protein contains an extra helix (α4) at the C-terminus and that this helix is of functional importance in CsrA binding. We therefore believe the dimer form B of CesT represents a biologically relevant form at least in physiological niches of interacting with CsrA and effector secretion. It remains unknown, however, if the chaperone molecule interacts with its effectors via dimer form A or form B, which should be studied in the future.

CsrA is a widely-distributed post-transcriptional regulator in gram negative bacteria. Its regulatory function is canonically modulated by sRNAs of high CsrA binding avidities (e.g., the CsrB/C and RsmX/Y/Z family members)^19–22,42^. These sRNAs will occupy the RNA binding site in CsrA, thereby competitively inhibiting the interactions between CsrA and mRNAs^23,26,27^. Although the sRNA-dependent CsrA modulation has been comprehensively studied, the regulation of CsrA activity by a protein ligand is only emerging in recent years. A previous study reported the structure of *Geobacillus thermodenitrificans* CsrA bound with FliW^43^, which revealed that FliW allosterically antagonizes CsrA in a non-competitive manner. CesT is a newly identified proteinaceous CsrA modulator in EPEC and EHEC. In contrast to FliW, our structure shows that CesT binds to CsrA at a site that extensively overlaps with that for RNA engagement, justifying again a competitive modulation mode as with sRNAs. CesT is therefore mechanistically bearing a much more resemblance to sRNAs than to the protein ligand of FliW.

In comparison to sRNAs with up to 10 nM affinity^27^, however, CesT represents a relative low-affinity CsrA-ligand (148 nM). We noted that despite the competitions, obvious inhibition of the CsrA/RNA interactions by CesT was only observed when CesT concentration is high (e.g., with two fold excess of CesT or higher) but not when the CesT/CsrA molar ratio is 1:1 (Fig. 5b), echoing the relatively-low affinity between CesT and CsrA. It is notable that CsrA and its regulatory sRNAs (CsrB and CsrC) form an auto-regulatory circuitry in EHEC and EPEC, enabling the system to be accurately modulated^19, 33^. CesT as a T3SS chaperone protein, however, is not involved in and thereby modulated by this Csr circuitry. In such a context, we believe the relatively low-binding affinity between CesT and CsrA is likely a biological way of enabling EHEC and EPEC to respond only after the cell-surface attachment of the bacteria when high concentration of CesT is available after effector secretion. It is also noteworthy that both CesT and CsrA created two ligand binding sites in their homodimers, which can lead to complex polymerization and subsequently the formation of higher aggregates in solution via sequential stacking of the two protein dimers. It is interesting that our comparative RMSA analyses showed that the inhibition of CsrA/RNA binding by CesT is more effective when with WT CsrA than with Re-CsrA, revealing that CesT/CsrA polymerization represents a contributing factor that can benefit CsrA repression by CesT (Supplementary Fig. 6). We note the very similar binding affinities between WT CsrA (148 nM) and Re-CsrA (151 nM) to CesT, the increased repression efficiency observed with WT CsrA could therefore indicate a possible cooperative polymerization process between CesT and CsrA. We believe that this might represent an efficient way of sequestering CsrA by CesT to enable the bacteria to quickly adapt to life on the epithelium surface.

## Methods

### Protein expression and purification

The *CesT* and *CsrA* genes of the EPEC O127:H6 E2348/69 strain (accession number: NC_011601.1) were synthesized by the GENEWIZ corporation (codon optimized for *E. coli*). For CesT (residues 1–156) and its variants including CesT-C_del_ (residues 1–145), CesT-E121A (residues 1–156, E121A), CesT-Y152A (residues 1–156, Y152A), and CesT-E121A/Y152A (residues 1–156, E121A + Y152A), the DNA fragments were first engineered to contain a coding sequence for an N-terminal 6xHis tag and a PreScission Protease (PSP) cleavage site, and then subcloned into the pET-30a (Novagen) vector via the NdeI and XhoI restriction sites. For CsrA (residues 1–61), CsrA-C_del_ (residues 1–44), Re-CsrA (CsrA-C_del_ linked to full-length CsrA via a GGGGSGGGGS linker), and the CsrA tetra-mutant (residues 1–61, R31A + K38A + H43A + R44A), the coding sequences were individually constructed into the pGEX-6P-1 (GE Healthcare) vector via the BamHI and XhoI restriction sites. The primers used for plasmid constructions were summarized in Supplementary Table 1. The resultant recombinant plasmids were then transformed into *E. coli* BL21(DE3) for protein expressions, which were conducted with the addition of 500 μM isopropyl-β-d-thiogalactoside (IPTG) followed by induction at 16 °C for ~12 h.

For protein purification, the *E. coli* cells were harvested, lysed by sonication in a re-suspension buffer composed of 20 mM Tris-HCl (pH 7.6) and 150 mM NaCl, and clarified via centrifugation at 18,000 × *g* for 20 min. For preliminary purifications, the CesT proteins were passed through the Ni-nitrilotriacetic acid (Ni-NTA) column (GE Healthcare) first, washed with the re-suspension buffer containing 20 mM imidazole, and then eluted with re-suspension buffer supplemented with 200 mM imidazole. For the CsrA proteins, the clarified supernatant was passed through the GST column (GE Healthcare), washed with re-suspension buffer to remove contaminated proteins, and then eluted with re-suspension buffer containing 20 mM GSH. The eluted target proteins were then further purified by gel filtration in re-suspension buffer using a Hiload 16/60 Superdex 200 pg (GE Healthcare) column. For the proteins used for crystallization, the fusion tags were removed in advance by on-column PSP-digestions in re-suspension buffer supplemented with 1 mM DTT.

### In vitro GST pull-down assay

The GST-fused CsrA proteins (GST-CsrA and GST-CsrA-C_del_) and wild type CesT were individually purified as described above. For the pull-down experiments, 60 μg GST or GST-CsrA or GST-CsrA-C_del_ was first incubated with 200 μg CesT for 2 h at 4 °C. The samples were then loaded onto GST-resin columns, followed by washing with PBST to remove the unbound proteins. The bound protein species were then analyzed by SDS-PAGE on a 12% gel and stained with Coomassie blue.

### SPR assay

The SPR analyses were carried out using BIAcore X-100 system with the NTA chips (GE Healthcare) at 25 °C. For the SPR measurements, all the proteins, including CesT, CsrA, and Re-CsrA, were prepared in a PBST buffer consisting of 1× PBS (136 mM NaCl, 2.6 mM KCl, 8 mM Na_2_HPO_4_, 2 mM KH_2_PO_4_, pH 7.4) and 0.005% Tween 20. The CesT protein was immobilized on the Ni-NTA chip at ~50 response units. The blank channel served as the negative control. When the data collection was finished in each cycle, the sensor surface was regenerated with 350 mM EDTA and then with 0.5 mM NiCl_2_. Gradient concentrations of CsrA or Re-CsrA ranging from 16 nM to 256 nM were flowed over CesT on the chip-surface at 30 μl/min, and the amount of bound proteins in response units was recorded for comparison.

The kinetic analyses were analyzed with BIAevaluation software 4.1. For dissociation constant (*K*_D_) calculations, the data of CsrA binding to CesT were fitted to a Bivalent-Analyte binding model, whereas the data of Re-CsrA binding to CesT were fitted to a Steady-State Affinity model.

### Analytical gel filtration chromatography

Purified CsrA and Re-CsrA were individually mixed with CesT with a molar ratio of 1.2:1, followed by incubation at 4 °C for 2 h. The samples, including the individual CsrA, Re-CsrA, and CesT proteins, as well as the CesT/CsrA and CesT/Re-CsrA mixtures, were loaded onto a calibrated Superdex 200 Increase 10/300 GL (GE Healthcare) column, respectively. The UV-280 curves were recorded and overlaid. The pooled samples were further analyzed by SDS-PAGE (15% gel) and stained with Coomassie blue.

For CesT and CsrA mutants, the purified proteins and their respective wild-type proteins were individually loaded on the Superdex 200 Increase 10/300 GL (GE Healthcare) column for comparative analytical gel filtration analyses. The UV-absorbance curves were recorded at 280-nm for all the proteins except for CsrA-C_del_, whose absorbance chromatograph was recorded at 215-nm due to the lack of any aromatic residues after deletion of the C-terminal helix. The curves were then overlaid for comparison.

### Crystallization and data collection and processing

For crystallization experiments, the Re-CsrA and CesT proteins were first mixed at 1:1 molar ratio and then further purified via gel filtration on a Hiload 16/60 Superdex 200 pg (GE Healthcare) column to obtain stable CesT/Re-CsrA complex. The resultant protein complex was used to screen the commercial crystallization kits (Hampton Research) via the sitting drop vapor diffusion method. The conditions were then optimized. Diffractable crystals were obtained by mixing 1 μl of the protein complex (at 10 mg/ml) with 1 μl reservoir solution consisting of 20% PEG 3350 and 0.2 M sodium citrate tribasic dihydrate, pH 8.3, followed by incubation at 18 °C for about 1 week. Data collection were conducted at Shanghai Synchrotron Radiation Facility (SSRF) beamline BL18U1. The collected data were then processed with HKL2000^44^ for indexing, integration, and scaling.

### Structure determination

The CesT/Re-CsrA structure was determined by the molecular replacement method using the Phaser program^45^ in the CCP4 suite^46^. The previously reported structures of CesT (PDB code: 1K3E) and CsrA (PDB code: 2BTI) were used as the search models. For CesT, the model structure was first truncated to include only residues I36-G131 prior to molecular replacement. The remaining CesT amino acids, including those consisting of the N-terminal α1-loop–β1 module and the extended C-terminal motif, were then manually rebuilt in Coot during refinement. For refinement, initial restrained rigid-body refinement was performed using REFMAC5^47^, which was followed by manual rebuilding and adjustment in COOT^48^. Further refinement was carried out with Phenix^49^. The stereochemical quality of the final model was assessed through the program PROCHECK^50^. The final data processing and structure refinement statistics are summarized in Table 1. All structural figures were generated using Pymol (http://www.pymol.org).

### Indirect ELISA

The indicated CsrA proteins, including CsrA, Re-CsrA, and the CsrA variants, were coated on 96-well plates (Corning) at 2 μg/well in 0.05 M carbonate–bicarbonate coating buffer overnight at 4 °C. The plates were then blocked for 1 h at 37 °C with 1× PBST containing 5% skim milk. Following blocking, serially-diluted His-tagged CesT proteins (wild type or variants) were added to the plates to bind to immobilized CsrA proteins for 1 h. The anti-his mAb (1:2000 diluted, mouse origin, Zen BioSicence, Cat. no. 230001) was then added to the wells and incubated for 1 h, followed by the addition of HRP-conjugated goat anti-mouse mAb (1:2000 diluted, Zen BioSicence, Cat. no. 511103) and incubation for about 40 min, and then the addition of TMB substrate and incubation for about 5 min at 37 °C. In each step, the plates were fully washed with PBST. The chromogenic reaction was then stopped with 2 M HCl and the emission OD450 was monitored using a microplate reader (Thermo).

### CD spectroscopy

Circular dichroism (CD) spectra were carried out on a CIRCULAR DICHRIOSM SPECTROMETER MODEL 400 (AVIV). Freshly prepared CesT (mutant and wild type) and CsrA (mutant and wild type) proteins were first adjusted to 0.1 mg/ml and 0.2 mg/ml in ultrapure water, respectively, and then applied for data collection. For each measure, wavelength spectra were recorded in the range of 190 nm to 260 nm at 25 °C using a 0.1-cm-path-length cuvette. Data was obtained by taking data points every 1 nm with a bandwidth of 1 nm.

### RNA gel mobility shift assay

The gel mobility shift assay was performed as previously described with small modifications^5^. In brief, an RNA molecular derived from *nleA* 5′UTR was synthesized with the sequence of 5′-CACUAAUAAUAUCAAUGGAUUGAUAUUAUUAAUG-3′ (Sangon Biotech). The RNA was dissolved in DEPC water, heated to 80 °C for 10 min, and slowly cooled down to room temperature before use. For the RNA-binding test, 1 μl pre-treated RNA solution (10 μM) was incubated, at room temperature for 30 min, with WT CsrA or the WT + C_del_ CsrA mixture or Re-CsrA at the indicated concentrations, and then resolved by 12% native TBE PAGE. For the experiments of CesT antagonizing the CsrA/RNA binding, decreasing concentrations of CesT were added to the pre-incubated CsrA/RNA (WT or WT + C_del_) or Re-CsrA/RNA binding reactions and then further incubated for 2 h at room temperature. The resultant reactions were then resolved using 12% native TBE PAGE. In each case, the gel was stained with ethidium bromide and the RNA bands were visualized with Gel DocTM XR + (BIO-RAD).

### Data availability

The coordinates and the related structure factors have been deposited into the Protein Data Bank with a PDB code of 5Z38 [http://www.rcsb.org/structure/5Z38]. The uncropped images of blots and gels presented in this paper are provided in Supplementary Figure 7. Other data are available from the corresponding author upon reasonable request.

## Electronic supplementary material

Supplementary Information(PDF 962 kb)
