# Supplementary material for: Molecular basis of binding between the global post-transcriptional regulator CsrA and the T3SS chaperone CesT
Source: Nat Commun. 2018 Mar 22;9:1196. doi: 10.1038/s41467-018-03625-x (PMC5864733; doi:10.1038/s41467-018-03625-x)
Supplement: Supplementary file 1 — Supplementary Information(PDF 962 kb) [file 41467_2018_3625_MOESM1_ESM.pdf]

Molecular basis of binding between the global post-transcriptional regulator CsrA and the T3SS chaperone CesT

Ye et al.

Supplementary Information

Supplementary Figure 1.

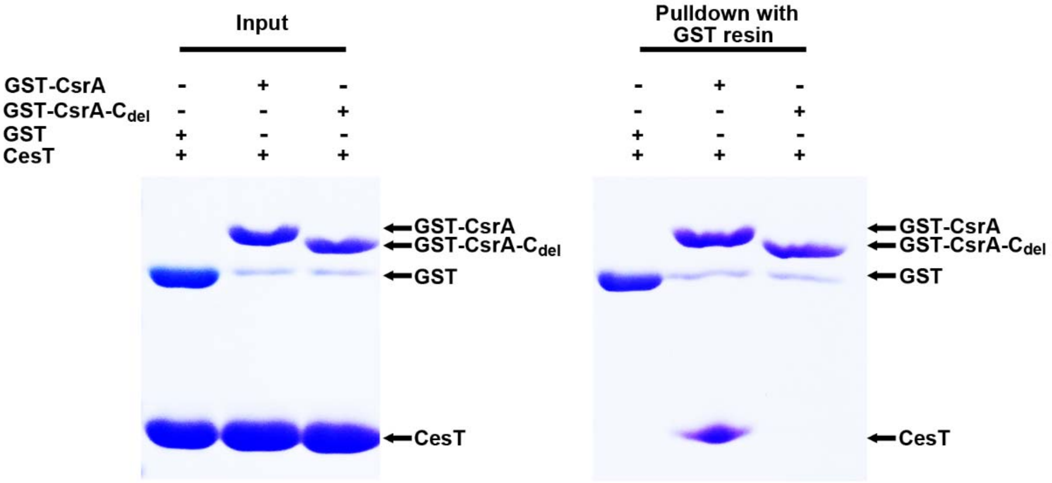

Supplementary Figure 1. A GST-pulldown assay showing the indispensable role of CsrA C-helix in the CesT/CsrA engagement. The purified GST-CsrA or GST-CsrA-C_{del} or GST proteins were individually incubated with CesT and then pulled down with GST-resins. The pooled samples were analyzed by SDS-PAGE and stained with coomassie blue. The migration profiles are shown.

Supplementary Figure 2.

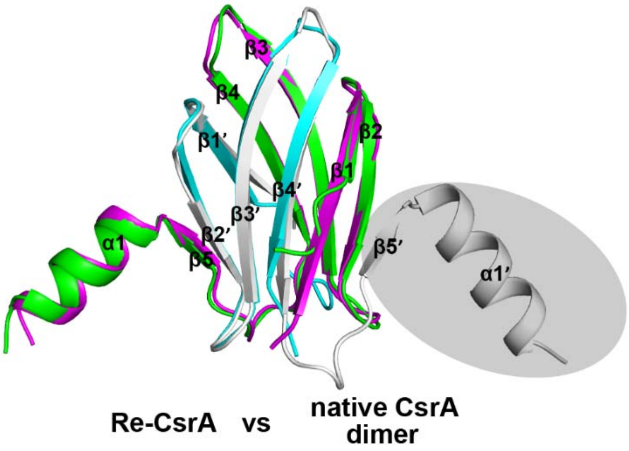

Supplementary Figure 2. Structural comparison of the native CsrA dimer with Re-CsrA. The two protomers in the native CsrA dimer structure are colored magenta and gray, respectively; while the CsrA-C_{del} and the full-length CsrA moieties in Re-CsrA are shown in cyan and green, respectively. The β5′ strand (which is untraceable in CsrA-C_{del}) and the α1′ helix (which is absent in CsrA-C_{del}) are shaded.

Supplementary Figure 3.

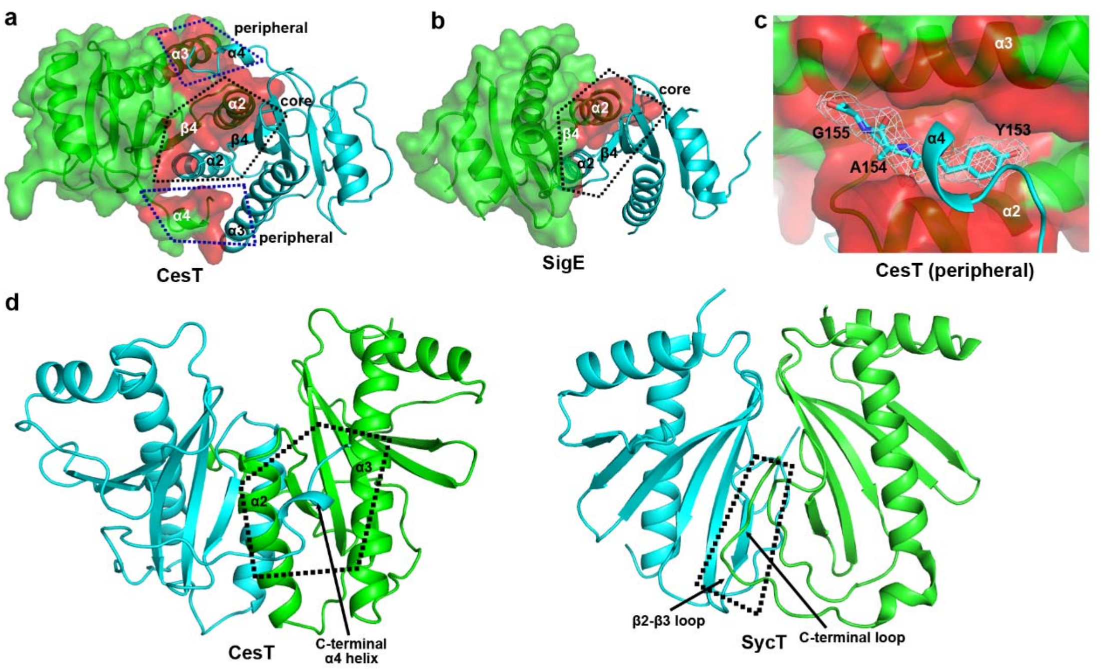

Supplementary Figure 3. A dimeric CesT architecture with a novel swapped C-terminal α-helix. **(a).** The form B dimeric structure of CesT. The two protomers are respectively shown in surface and cartoon representations, with their dimer interface being highlighted in red. The core (which is created by the stacked α2 helices and β4 strands) and peripheral (which is formed by the swapped α4 helix interacting with helices α2 and α3) interfaces are encircled with dashed black- and blue-lines, respectively, and are labeled. **(b).** The unswapped dimeric structure of salmonella SigE (PDB code: 1K3S). The molecule only contains a core dimeric interface, which is highlighted and labeled. **(c).** A magnified view of the peripheral dimeric interface in CesT. The color scheme is the same as in panel **a**. Those residues that protrude into the α2/α3 groove are highlighted and labeled. The electron densities are contoured at 1.0 σ above the mean using the 2|Fo|-|Fc| map. **(d).** Comparison of the SycT (PDB code: 2BSJ) and CesT (form B) dimer structures highlighting their extended C-termini. For each structure,

the two molecules in the dimer are colored green and cyan, respectively. The C-terminal components and their interaction pairs in the other protomer are encircled using dashed lines and labeled. Left panel, dimeric structure of CesT; Right panel, dimeric structure of SycT.

Supplementary Figure 4.

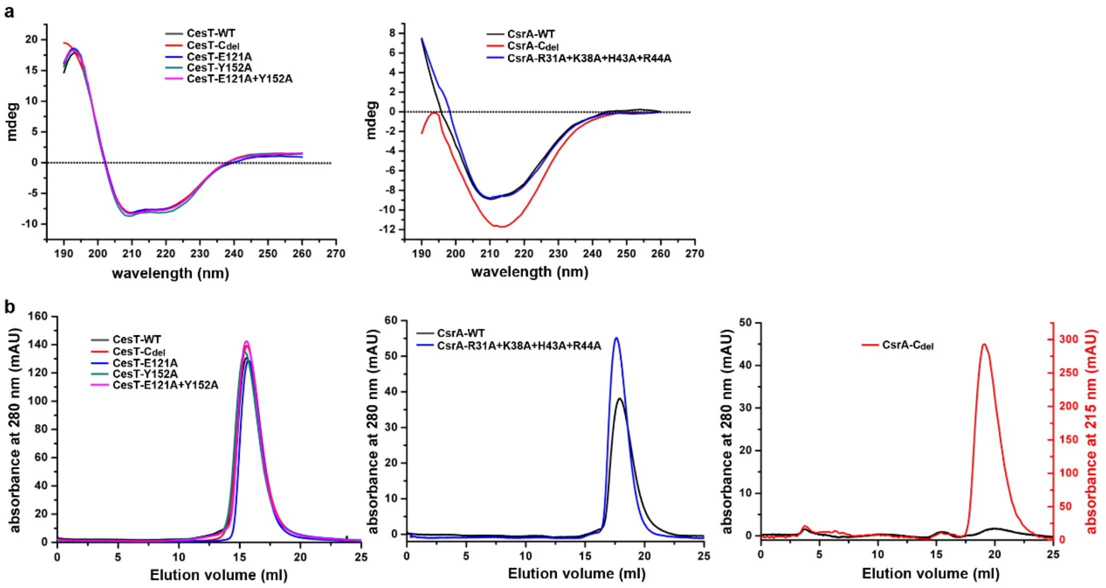

Supplementary Figure 4. CD spectroscopic and comparative analytical gel-filtration studies on CesT and CsrA mutants. **(a).** CD spectra of the wild type and mutant CesT (left panel) and CsrA (right panel) proteins. Clearly shown is that the observed CD-profiles of all the CesT mutants are quite similar to that of wild type CesT and the CsrA tetra-mutant to wild type CsrA, demonstrating their proper folds as the native proteins. For CsrA-C_{del}, deletion of the C-terminal helix would consequently leave a single core β-barrel. Consistently, the recorded CD spectrum of CsrA-C_{del} indeed shows features of β-structures, indicating its proper folding. **(b).** Analyses of the wild type and mutant CesT (left panel) and CsrA (middle panel for wild type CsrA and CsrA tetra-mutant, and right panel for CsrA-C_{del}) proteins by comparative analytical gel-filtration. All the CesT mutants were eluted in the same position as wild type CesT and CsrA tetra-mutant as wild type CsrA, demonstrating their dimeric states in solution. For CsrA-C_{del}, the UV-absorbance was monitored at 215-nm because this deletion mutant contains no aromatic amino acids. The profile revealed a delayed elution volume in comparison to

the wild type or the tetra-mutant proteins, which is consistent with its reduced molecular size (by ~1/3 of the molecular mass of the wild type protein) after deletion of the C-terminal helix.

Supplementary Figure 5.

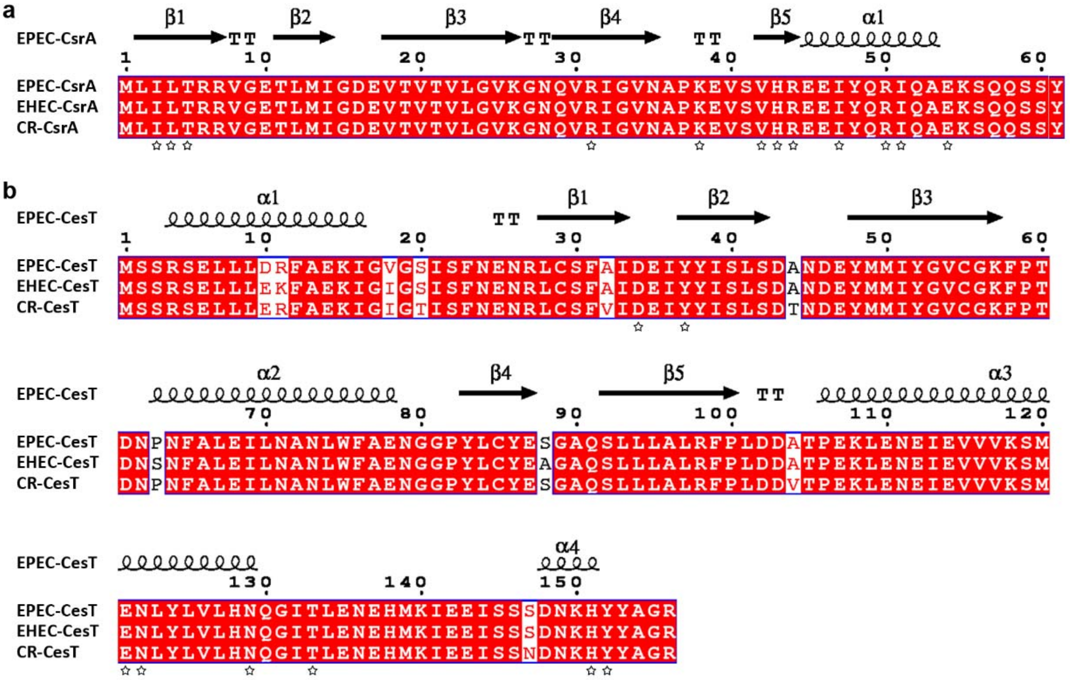

Supplementary Figure 5. Structure-based sequence alignments for the CsrA and CesT proteins derived from EPEC, EHEC, and *Citrobacter rodentium* (CR). The spiral lines represent α-helices, while the horizontal arrows indicate β-strands. The identified interface residues are highlighted with hollow stars. **(a).** Comparison of the CsrA sequences **(b).** Comparison of the CesT sequences. Accession codes: EPEC-CsrA, CAS10507.1; EHEC-CsrA: AAG57800.1; CR-CsrA: CBG89847.1; EPEC-CesT, CAS11488.1; EHEC-CesT, AAG58824.1; CR-CesT, CBG89717.1.

Supplementary Figure 6.

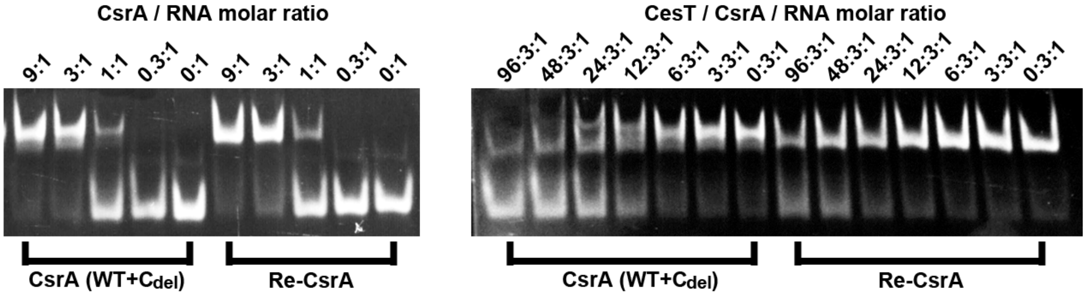

Supplementary Figure 6. A comparative RMSA experiment using the CsrA WT+C_{del} mixture (WT CsrA mixing with CsrA-C_{del} in a 1:1 molar ratio) and Re-CsrA. The RNA molecule was incubated with the indicated CsrA species (WT+C_{del} or Re-CsrA) in the absence or presence of gradient concentrations of CesT and then analyzed by native TBE PAGE. The stained gels are shown. Left panel: WT+C_{del} CsrA-mixture and Re-CsrA mixing with RNA at the indicated molar ratios. Right panel: pre-incubated CsrA/RNA mixtures (WT+C_{del} plus RNA and Re-CsrA plus RNA) at 3:1 molar ratio with different concentrations of CesT.

Supplementary Figure 7.

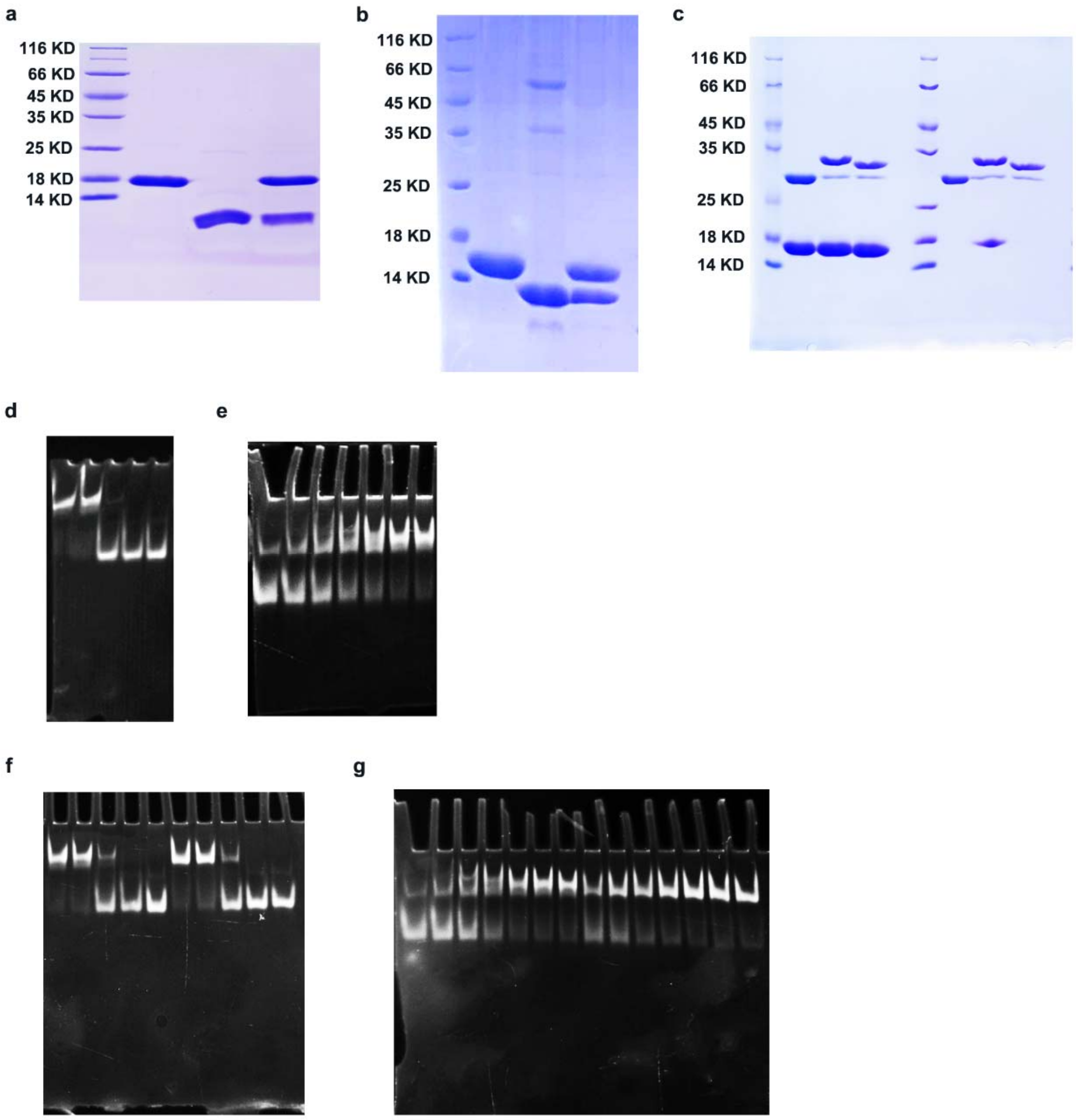

Supplementary Figure 7. Uncropped images of blots and gels in this paper. **(a, b).** Original SDS-PAGE images presented in Figure 1. **(c).** Original SDS-PAGE images presented in Supplementary Figure 1. **(d, e).** Original native TBE-PAGE gel-images presented in Figure 5. **(f, g).** Original native TBE-PAGE gel-images presented in Supplementary Figure 6.

Supplementary Table 1 Primers used for plasmid constructions

Name	Sequence	Usage
CesT_FP2	GGAATTCCATATGCACCATCATCACCAT	Creation of the CesT, CesT-C_{del}, CesT-E121A, CesT-Y152A, CesT-E121A/Y152A expression plasmids, priming with CesT_RP, CesT_C_{del}_RP, CesT_E121A_RP1, CesT_Y152A_RP, respectively.
CesT_FP1	CACCATCATCACCATCATCTGGAAGTGCTGTTTCAGGGTCCGATGAGCAGCCGCAGC	Creation of the CesT expression plasmid (adding N-terminal 6xHis and PSP cleavage site), priming with CesT_RP.
CesT_RP	CGCTCGAGTTAGCGACCGGC	Creation of the CesT, CesT-E121A expression plasmids.
CesT_C_{del}_RP	CGCTCGAGTTAGCTAATTTCTTCAATTTTC	Creation of the CesT-C_{del} expression plasmid.
CesT_E121A_RP1	CACCAGATACAGGTTCGCCATGCTTTTAACAAC	Creation of the CesT-E121A, CesT-E121A/Y152A expression plasmids.
CesT_E121A_FP2	GTTGTTAAAAGCATGGCGAACCTGTATCTGGTG	Creation of the CesT-E121A, CesT-E121A/Y152A expression plasmids, priming with CesT_RP.
CesT_Y152A_RP	CGCTCGAGTTAGCGACCGGCATAAGCATGTTTATTATC	Creation of the CesT-Y152A, CesT-E121A/Y152A expression plasmids.
CsrA_FP	AAGGATCCATGCTGATTCTGAC	Creation of the CsrA, CsrA-C_{del}, Re-CsrA, CsrA tetra-mutant expression plasmids, priming with CsrA_RP, CsrA_1-44_RP, Re-CsrA_RP1, CsrA_RKHR/A_RP1, and CsrA_RKHR/A_RP2, respectively.
CsrA_1-44_RP	CGCTCGAGTTAGCGATGCACGCTCAC	Creation of the CsrA-C_{del} expression plasmid.
Re-CsrA_RP1	TGAGCCTCCTCCTCCAGATCCGCCTCCGCCGCGATGCACGCTCAC	Creation of the Re-CsrA expression plasmid.
Re-CsrA_FP2	GGCGGAGGCGGATCTGGAGGAGGAGGCTCAATGCTGATTCTGAC	Creation of the Re-CsrA expression plasmid, priming with CsrA_RP.
CsrA_RP	CGCTCGAGTTAATAGCTGCTCTG	Creation of the CsrA and Re-CsrA expression plasmids.
CsrA_RKHR/A_RP1	CTCGGCAGCCACGCTCACTTCTGCCGGGGCATTCACGCCAATGGCCACCTGATTGCCTT	Creation of the CsrA tetra-mutant expression plasmid.
CsrA_RKHR/A_RP2	CGCTCGAGTTAATAGCTGCTCTGCTGGCTTTTTTCGGCCTGAATGCGCTGATAGATTTCCTCGGCAGCCACGCT	Creation of the CsrA tetra-mutant expression plasmid.

Primers used in this study. Underlined letters represent the restriction sites inserted.
